# The Application of Anisotropically Collapsing Gels, Deep Learning, and Optical Microscopy for Chemical Characterization of Nanoparticles and Nanoplastics

**DOI:** 10.1021/acs.langmuir.5c00769

**Published:** 2025-05-19

**Authors:** Hana Brožková, Julie Weisová, Antonín Hlaváček

**Affiliations:** † Department of Chemistry, Faculty of Science, Masaryk University, 625 00 Brno, Czech Republic; ‡ Institute of Analytical Chemistry of the Czech Academy of Sciences, Veveří 97, 602 00 Brno, Czech Republic

## Abstract

The surface chemistry of nanomaterials, particularly the density of functional groups, governs their behavior in applications such as bioanalysis, bioimaging, and environmental impact studies. Here, we report a precise method to quantify carboxyl groups per nanoparticle by combining anisotropically collapsing agarose gels for nanoparticle immobilization with fluorescence microscopy and acid–base titration. We applied this approach to photon-upconversion nanoparticles (UCNPs) coated with poly­(acrylic acid) (PAA) and fluorescence-labeled polystyrene nanoparticles (PNs), which serve as models for bioimaging and environmental pollutants, respectively. UCNPs exhibited 152 ± 14 thousand carboxyl groups per particle (∼11 groups/nm^2^), while PNs were characterized with 38 ± 3.6 thousand groups (∼1.7 groups/nm^2^). The limit of detection was 6.4 and 1.9 thousand carboxyl groups per nanoparticle, and the limit of quantification was determined at 21 and 6.2 thousand carboxyl groups per nanoparticle for UCNP-PAAs and PNs, respectively. High intrinsic luminescence enabled direct imaging of UCNPs, while PNs required fluorescence staining with Nile Red to overcome low signal-to-noise ratios. The study also discussed the critical influence of nanoparticle concentration and titration conditions on the assay performance. This method advances the precise characterization of surface chemistry, offering insights into nanoparticle structure that extend beyond the resolution of electron microscopy. Our findings establish a robust platform for investigating the interplay of surface chemistry with nanoparticle function and fate in technological and environmental contexts, with broad applicability across nanomaterials.

## Graphical abstract

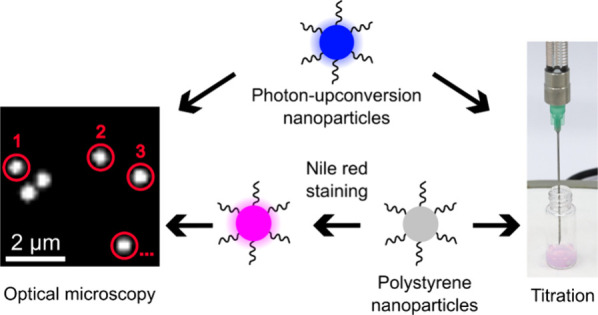

## Introduction

Nanomaterials present many applications in life and material sciences, including catalysis, bioimaging, and drug delivery.^1–7^ Their use in practice is governed by their physicochemical properties, such as size, shape, structure, chemical composition, and others. On the other hand, nanomaterials such as nanoplastics are spreading into the environment, and control is possible with only limited analysis tools.^8–13^ An important property is the chemistry of nanoparticle surfaces.^14,15^ The number of functional groups controls colloidal stability, biocompatibility, toxicity, and interactions with other molecules and nanoparticles, and enables conjugation to ligands and biomolecules.^16–18^ The estimation of the average number of functional groups per single dispersed nanoparticle is generally made in two steps. First, the molar concentration of functional groups in the dispersion is estimated. Second, the number or molar concentration of nanoparticles needs to be found (note that number and molar concentrations are the same quantities only differing in name and commonly used unit^19,20^ ). The ratio of these two quantities estimates the average number of functional groups per nanoparticle.

One can use a wide range of analytical methods to quantify functional groups.^14^ On the other hand, the quantification of nanoparticles is underdeveloped. Available techniques measure either the ensemble property of nanoparticle dispersion or utilize nanoparticle counting.^19^ In ensemble methods, a piece of prior knowledge is needed for estimating the concentration of nanoparticles. For instance, the absorbance of dispersion is measured as an ensemble property (i.e., as an analytical signal). A calibration curve then relates the absorbance values with the calibration standards’ concentrations. The concentration of the unknown sample is then calculated based on the calibration curve. In contrast, the counting approaches are straightforward and do not rely on prior knowledge. The molar or number concentration of nanoparticles is estimated by counting the nanoparticles occupying a given sample volume. Two types of counting approaches may be recognized. In serial counting, the nanoparticles are counted one by one when a stream of nanoparticle dispersion is focused in a channel through a nanoparticle detector. Depending on nanoparticle type, optical detection,^21^ resistive pulse sensing,^22^ or mass spectroscopy^23^ is used to count the nanoparticles. In a parallel setting, microscopy is used for imaging and counting large numbers of nanoparticles at once in a single micrograph.^24^ For instance, optical microscopy is used for counting and even tracking single nanoparticles. This technique works well for particles providing a high enough signal. As particles get smaller, the background signal of solvents such as fluorescence, and Raman or Rayleigh scattering eventually hide the signal from nanoparticles in noise. This phenomenon ultimately prevents their imaging and counting through optical microscopy.^25^ Indeed, the removal of solvent molecules is also a prerequisite for utilizing electron microscopy (unless an ultrathin liquid cell is available^26^). To overcome this limitation, specialized sampling approaches are currently being developed when the nanoparticles are immobilized on a suitable substrate and the solvent is removed traceably—without losing the information on the sampled volume. For instance, a so-called “nanopipette” was developed for traceable removal of solvent molecules for transmission electron microscopy (TEM). For optical microscopy, we recently introduced a method of anisotropically collapsing gels.^27^ In this method, nanoparticles are mixed with melted agarose in water and cast as a microlayer of an agarose gel of precisely known thickness. After evaporating water from the gel microlayer, the gel structure collapses anisotropically—its thickness is significantly reduced but lateral dimensions remain unchanged. The immobilized nanoparticles are imaged with extreme sensitivity, and multiplexed imaging is feasible.

Here, we apply the method of anisotropically collapsing gels to estimate the average number of functional groups on nanoparticle surfaces. The carboxyl groups were selected as a model functional group for their wide application in bioconjugation chemistry and their influence on the fate of microplastics and nanoplastics in the environment.^14,28,29^ Acid–base titration with colorimetric detection of equivalence points was used for their quantification. As model nanoparticles, we utilized photon-upconversion nanoparticles coated with poly­(acrylic acid) (UCNP-PAAs) for their excellent applicability for biological imaging and high signal-to-background ratio (Figure 1a). The second model—polystyrene nanoparticles coated with poly­(acrylic acid) (PNs)—was utilized as a model of environment pollution nanoplastics.^9^ Additionally, PNs are models of nanoparticles with a low signal-to-background ratio, which needs fluorescence staining before counting them (Figure 1b).^30,31^ This was performed by soaking the NPs with the fluorescent dye Nile Red. Finally, the average number of carboxyls was estimated for each nanoparticle type and discussed in the context of nanoparticle size, structure, and analytical characteristics.

**Figure 1:**
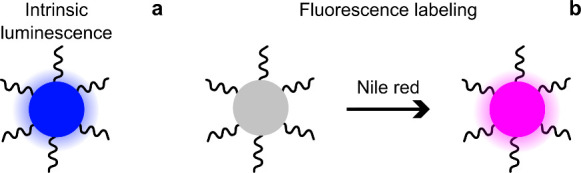
Scheme of studied nanoparticles. (a) UCNP-PAAs provide intrinsic luminescence (blue coloration, excitation 976 nm, emission 802 nm), (b) Intrinsically nonluminescent PNs are soaked with Nile red to become fluorescent (pink coloration, excitation 520 nm, emission 560 nm). In panels (a) and (b), the ribbons represent PAA chains on the nanoparticle surfaces.

## Experimental Section

### Materials

Oleic acid-capped photon-upconversion nanoparticles (UCNPs, NaYF_4_/18% Yb^3+^/2% Tm^3+^, hydrodynamic diameter 66 nm, 48 mg mL^–1^) and polystyrene nanoparticles coated with poly­(acrylic acid) (PNs, hydrodynamic diameter 83 nm, 90 mg mL^–1^) were prepared as reported previously with minor modifications.^27,32,33^ Polyacrylic acid (PAA, average molecular weight 100 kg mol^–1^, 35 wt %), Tween 20, *N*-hydroxysulfosuccinimide sodium salt (Sulfo-NHS), 2-(*N*-morpholino)­ethanesulfonic acid monohydrate (MES), nitrosyl tetrafluoroborate, and Methyl Orange were purchased from Sigma–Aldrich/Merck (Germany). Nile Red dye, tris­(hydroxymethyl)­aminomethane, and agarose NEEO ultra-Qualität were obtained from Carl Roth (Germany); oxalic acid dihydrate, phenolphthalein, and calcium chloride anhydrous were obtained from LACHEMA (Czech Republic); sodium hydroxide, dimethylformamide, and cyclohexane were obtained from PENTA (Czech Republic); 1-ethyl-3-(3-(dimethylamino)­propyl)­carbodiimide (EDC) was obtained from Fluka/Merck (Germany); and tetrahydrofuran (THF) was obtained from Lach-Ner (Czech Republic).

### Modification of UCNPs with Poly­(acrylic acid)

According to the previously reported method,^34^ oleic acid-capped UCNPs in cyclohexane (10 mg, 200 μL) were mixed with NOBF_4_ in DMF (0.35 mg, 3 μmol, 300 μL) at room temperature for an intermediate ligand exchange. The resulting mixture was vigorously stirred for 10 min. The UCNPs were transferred from the upper cyclohexane layer to the layer of DMF and sedimented by centrifugation (13 000 G, 10 min). The surface-modified UCNPs were washed with 1 mL of DMF and centrifuged (13 000 G, 10 min). The pellet was redispersed in 1 mL of deionized water. For ligand exchange, 1 mL of the water-dispersed UCNPs was combined with 200 μL of aqueous poly­(acrylic acid) (80 mg mL^–1^), previously titrated to pH 9 by 1 M NaOH. The dispersion was sonicated for 5 min and then centrifuged (13 000 G, 10 min). The pellet was dispersed in 1 mL of deionized water, and the resulting dispersion of UCNP-PAAs was sonicated for 15 min and sedimented by centrifugation for 10 min at 13 000 G (repeated 4 times). Washed nanoparticles were then filtered by a nylon syringe filter (0.45 μm) and slow centrifugation (85 G; 75 min) to remove large clusters. A MiniSpin plus centrifuge (Eppendorf) was used for this procedure.

### Staining Polystyrene Nanoparticles with Nile Red

A mixture of 16.5 μL of Nile Red solution in THF (1 mmol L^–1^) and 100 μL of an aqueous solution of polystyrene nanoparticles (90 mg mL^–1^) was incubated for 15 min.^35^ After adding 133 μL of water, the resulting dispersion was centrifugated (20 000 G, 60 min). The pellet was then subjected to centrifugation three times and washed with 500 μL of water (20 000 G, 60 min). Centrifuge 5804R (Eppendorf) was used for this procedure.

### Counting Assay for Estimating Nanoparticle Molar Concentration

As described previously,^27^ samples were mixed with melted agarose (1.25% w/w, 60 °C) and cast onto a glass slide (1 mm thick) with plastic tape spacers to set the layer thickness to 38 ± 1.2 μm (mean ± standard deviation, *n* = 10). The agarose dispersion was covered with a second glass slide (1 mm thick) and secured with clips. After gelling in a refrigerator, the top slide was removed at room temperature, allowing rapid water evaporation and anisotropic collapse of the agarose into a submicrometer layer (Figures 2a–d). Nanoparticles immobilized in the agarose layer were imaged using optical microscopy, localized via a convolutional neural network, and counted. The molar concentration (*c*) was calculated using eq 1:

1 $$c=\frac{N}{hw^{2}N_{A}}RD$$

where *N* is the number of nanoparticle spots, *h* is the gel thickness before collapse, *w* is the micrograph width, *N*_A_ is Avogadro’s number, *D* is the sample dilution, and *R* is a correction factor, which accounts for multiple nanoparticles contributing to a single spot and is determined from the histogram of spot intensities as previously described.^27^

**Figure 2:**
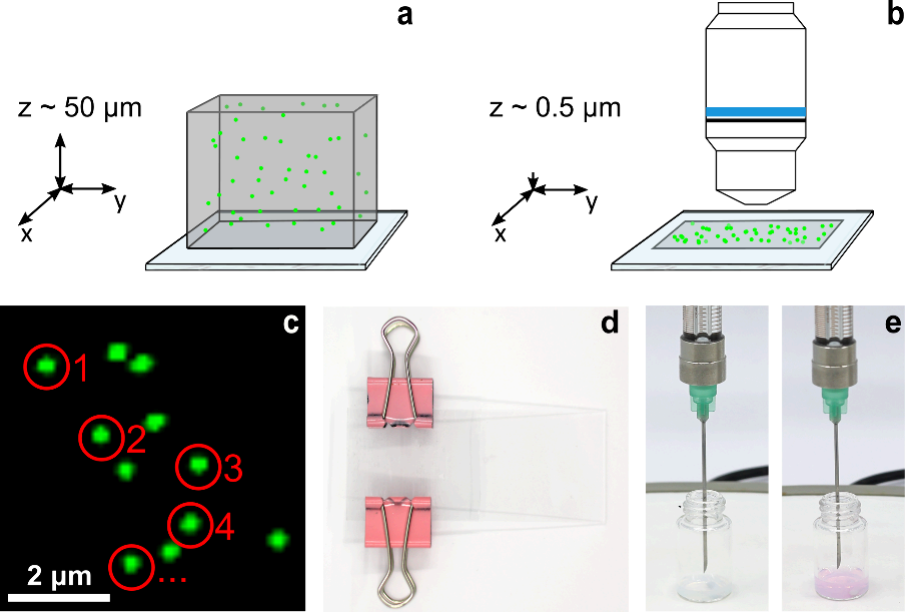
Nanoparticle counting assay based on anisotropic agarose gel collapse and acid–base titration. (a) Nanoparticles in aqueous dispersion are mixed with melted agarose and cast into a thin microlayer. (b) Upon air drying, the agarose collapses anisotropically into a submicrometer layer, imaged by optical microscopy. (c) Artificial intelligence algorithms are employed to count the nanoparticles. (d) Photograph of the agarose casting setup between two glass slides, secured with metal clips (spacer not visible). (e) Acid–base titration of nanoparticle samples using phenolphthalein as an indicator: before equivalence (left) and at the equivalence point (right).

### Optical Setup and Image Processing

Nanoparticle imaging was performed using a custom-built wide-field microscope with epiphoton-upconversion (UCNP-PAAs) and epifluorescence (Nile-PNs) modalities. For UCNP-PAAs, a 976-nm excitation laser was filtered through a 950 nm long-pass filter to remove shorter wavelengths and reflected by a 900 nm short-pass dichroic mirror into the sample through a microscope objective. Emissions were collected and passed through 875 nm short-pass and 800 ± 25 nm band-pass filters before projection onto the camera sensor using a 200 mm tube lens. For fluorescently labeled PNs, a 520-nm excitation laser was used with complementary filters (520 ± 20 nm band-pass for input, 550 nm long-pass dichroic mirror, and 650 ± 25 nm band-pass for emission). Micrographs were recorded using an iXon Life 888 EM-CCD camera. Micrographs were carefully calibrated to correct for illumination irregularities and remove instrumental background. Compared to photon-upconversion, fluorescence micrographs exhibited higher background noise. Automatic nanoparticle localization and counting were performed using a convolutional neural network with U-net architecture (see Note S1 and Scheme S1 in the Supporting Information).^27,36,37^

### Gel Electrophoresis

Gel electrophoresis was carried out in 0.75% agarose (Agarose NEEO ultraquality; Carl Roth, Germany) for 30 min with the electric voltage of 150 V. The electrophoresis buffer contained 50 mM tris­(hydroxymethyl)­aminomethane and 50 mM H_3_BO_3,_ resulting in a pH of 8.6. The sample solutions (18 μL) with an approximate concentration of 3 mg mL^–1^ of nanoparticles and a glycerol content of 5% were applied to each well. Gel images were acquired by a laboratory-built scanner in a photon-upconversion or light transmission mode (see Supporting Information Note S2 andScheme S2 in the Supporting Information).

### Quantification of Carboxyl Groups via Acid–Base Titration

Nanoparticles (7.2 mg UCNP-PAAs, 0.9 mg PNs) were washed twice with HCl (1 mmol L^–1^, 500 μL) by centrifugation (7000 G, 10 min; MiniSpin plus centrifuge, Eppendorf). After the final wash, the nanoparticle pellet was resuspended in HCl (1 mmol L^–1^, 100 μL), and the dispersion volume was measured using a pipet. Titration was performed in a vial using freshly prepared NaOH (2.5 mmol L^–1^) and phenolphthalein as an indicator, adding NaOH until a stable pink end point was achieved (Figure 2e). A blank titration of HCl (1 mmol L^–1^, 150 μL) without nanoparticles was conducted to account for NaOH consumption by HCl alone. The NaOH stock solution (100 mmol L^–1^) was standardized using oxalic acid dihydrate (50 mmol L^–1^) with Methyl Orange as the indicator. Upon color change to orange-yellow, calcium chloride (20% w/v) was added dropwise, restoring the pink color due to HCl release. The orange end point indicated equivalence.

## Results and Discussion

### Measurement Uncertainties

The combined uncertainty in the number of carboxyl groups per nanoparticle was derived from uncertainties in both nanoparticle counting and carboxyl group concentration measurements. We used a type B uncertainty estimate from our previous research to estimate the uncertainty associated with the nanoparticle counting assay in anisotropically collapsed agarose. As we reported previously,^27^ analyzing four samples revealed an average relative standard deviation of 8% between the counting assay and a reference method, which we adopted as the assay’s uncertainty in this study. Repeated experiments showed a relative standard deviation of 5%, suggesting that sources of uncertainty beyond repeatability, such as calibration of the agarose microlayer thickness and pipetting, contribute to the overall variability.^27^ In this study, the relative standard deviations for repeated measurements were 2% and 5% for UCNP-PAAs and PNs, respectively, confirming consistent assay performance across these experiments. For carboxyl group concentration measurements, the primary source of uncertainty was the repeatability of nanoparticle acid–base titration. The standard deviations from five replicate titrations were 2.3% for UCNP-PAAs and 3.1% for PNs. Additional contributions included pipet, syringe, and NaOH concentration calibrations, with relative standard uncertainties of 1%, 1%, and 1.5%, respectively. To calculate the combined uncertainties in carboxyl groups per nanoparticle, we employed a Monte Carlo simulation with 5 × 10^6^ random samples. The uncertainty calculations are summarized in Tables 1 and 2.

**Table 1:** Estimating the Number of Carboxyl Groups per Single UCNP-PAA^a^

	mean value	standard deviation	standard uncertainty
nanoparticle concentration (nmol L^–1^)	3.35	0.18^a^	0.27
carboxyl concentration (mmol L^–1^)	0.509	0.012^b^	0.019
number of carboxyls per nanoparticle	152 × 10^3^	–	14 × 10^3^

a The molar concentration of UCNP-PAAs was determined using the counting assay, and the concentration of surface carboxyl groups was quantified via acid–base titration. The average number of carboxyl groups per nanoparticle was calculated by dividing the carboxyl group concentration by the nanoparticle concentration.

b Standard deviation from two repeated agarose microlayers.

c Standard deviation from five repeated acid–base titrations.

**Table 2:** Estimating the Number of Carboxyl Groups Per Single PN^a^

	mean value	standard deviation	standard uncertainty
nanoparticle concentration (nmol L^–1^)	551	19​^a^	44
carboxyl concentration (mmol L^–1^)	20.84	0.​65^b^	0.98
number of carboxyls per nanoparticle	38 × 10^3^	–​	3.6 × 10^3^

a The molar concentration of PNs was determined using the counting assay, and the concentration of surface carboxyl groups was quantified via acid–base titration. The average number of carboxyl groups per nanoparticle was calculated by dividing the carboxyl group concentration by the nanoparticle concentration.

b Standard deviation from two repeated agarose microlayers.

c Standard deviation from five repeated acid–base titrations.

### Carboxylated Photon-Upconversion Nanoparticles

UCNPs represent intrinsically luminescent nanoparticles emitting a specific signal. UCNPs are advantageously used as detection labels in various diagnostic platforms, immunoassays, and sensors.^33,38,39^ Controlling the surface chemistry such as the number of functional groups—very often carboxyl groups—is critical for reproducibility in these applications. We prepared UCNPs composed of NaYF_4_ nanocrystals doped with Yb^3+^ (18%) and Tm^3+^ (2%). The UCNPs were coated from the synthesis with an oleic acid capping layer, stabilizing them in strongly hydrophobic solvents. They were characterized by 66 nm hydrodynamic diameter in cyclohexane (Figure 3a). A two-step ligand exchange process replaced the oleic acid with a poly­(acrylic acid) (PAA, molar mass 100 kg mol^–1^) to make nanoparticles water-dispersible. In the first ligand exchange step, the NOBF_4_ dispersed the nanoparticles in dimethylformamide, and the PAA was added and adsorbed on the nanoparticle surfaces in the next step. This created water-dispersible UCNP-PAAs with a hydrodynamic diameter of 92 nm (Figure 3a). The aqueous dispersion of UCNP-PAAs was stored in a refrigerator. The dispersion revealed an eye-visible blue emission (450 and 475 nm, Figures 3b and 3c) under the excitation of a 976 nm laser beam. Additionally, weak red (646 nm) and strong near-infrared (802 nm) peaks were present in the emission spectrum (Figure 3c).

**Figure 3:**
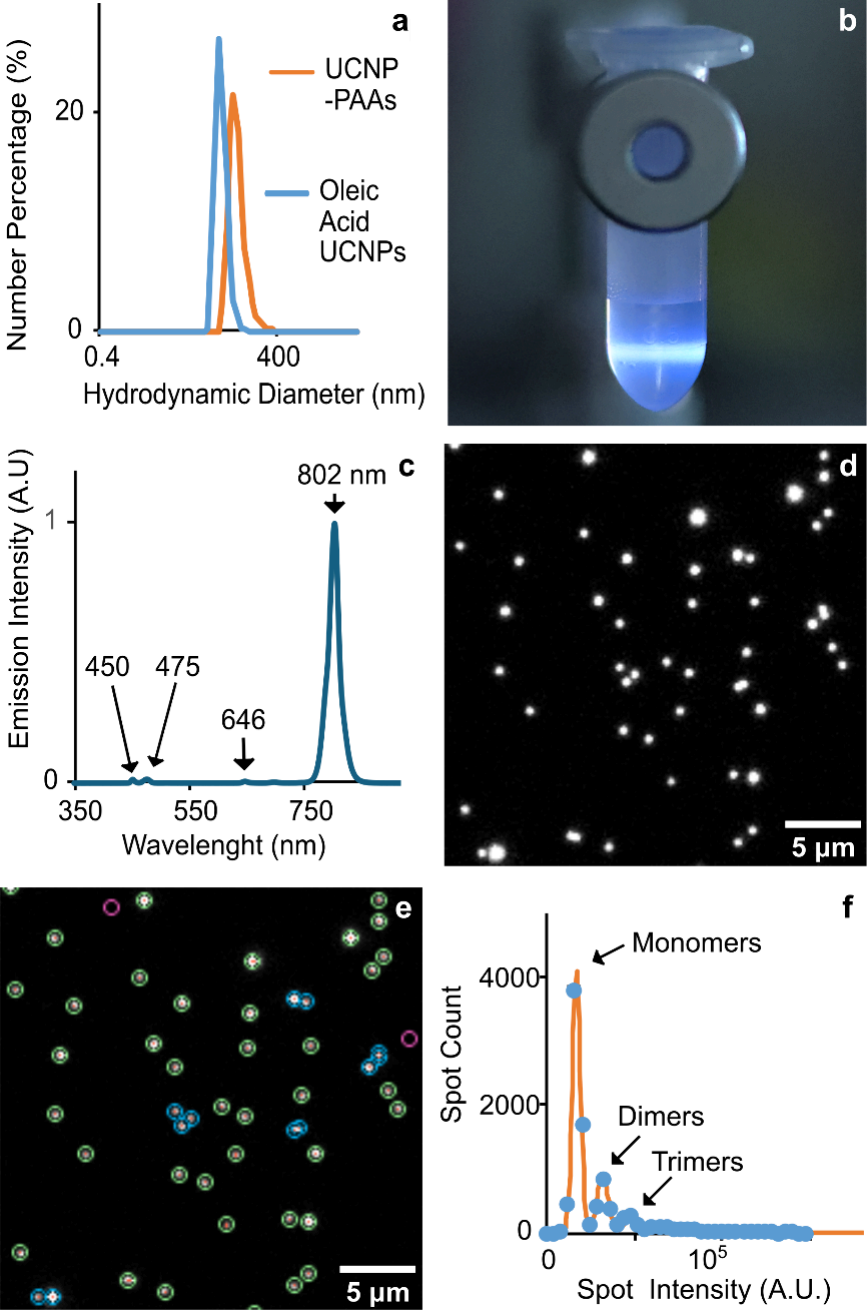
Characterization of photon-upconversion nanoparticles (UCNP). (a) Size distribution of nanoparticles with different surface modifications. (b) UCNP-PAAs emit visible blue light under a 976 nm laser beam. (c) Emission spectrum of UCNP-PAAs upon 976 nm excitation. (d) Photon-upconversion micrograph of UCNP-PAAs in collapsed agarose gel (976 nm excitation, 800 ± 25 nm emission, 3000 ms exposure). (e) Annotated version of panel (d): red dots indicate nanoparticle localizations identified by U-net; green circles represent measured spot intensities; blue circles mark spots excluded from intensity analysis; magenta circles indicate background signal placed sufficiently far from nanoparticles to avoid interference. (f) Spot intensity histogram fitted to a linear combination of three Gaussian curves, corresponding to monomers, dimers, and trimers of UCNP-PAAs.

### Analysis of Carboxylated Photon-Upconversion Nanoparticles

To estimate the nanoparticle’s molar concentration, UCNP-PAAs were diluted and immobilized in the agarose gel microlayer, which collapsed anisotropically after evaporating the water contained in the gel. The microlayers were prepared in two repeats and three micrographs were recorded for each microlayer preparation. UCNP-PAAs were observed as diffraction-limited spots in the epiphoton-upconversion imaging mode with an emission 800 ± 25 nm band-pass filter (Figure 3d). The utilized microscope objective (magnification 40×, numerical aperture 0.65) allowed for a large field of view covering the area of 300 μm × 300 μm. A convolution neural network of U-net architecture was used for spot localization (Figure 3e), and Python script analyzed their emission intensities (Figure 3f). The script measured the spot intensities and corrected them for a background signal. The measurement of close overlapping spots was omitted for possible interference between the spot signals and subsequent imprecision of spot intensity statistics. The background signal was measured in 100 random localizations within each micrograph but always far enough from nanoparticle spots. The histogram of spot intensities revealed three gradually decreasing maxima. These were fitted well by a linear combination of three Gaussian curves, as shown in Figure 3f. Based on our previous studies,^27^ these maxima can be interpreted as monomeric nanoparticles and their discrete aggregates of two or three UCNP-PAAs. This implies that the number of nanoparticles is higher than the number of spots. As described previously, a correction factor was calculated converting the number of spots to the number of nanoparticles. This correction factor was calculated at 1.66 from the ratios of areas below the fitted Gaussian curves. On average, 1344 spots were counted per single micrograph (∼15 thousand spots per mm^2^), which implies 2231 UCNP-PAAs per single micrograph after applying the correction factor (∼25 thousand UCNPs per mm^2^). Unfortunately, agarose gel electrophoresis could not confirm the presence of aggregates, likely due to the adsorption of UCNP-PAAs onto the agarose matrix (see Note S3 and Figure S1 in the Supporting Information). By considering the dilution and the thickness of the agarose microlayer before drying, the concentration of UCNP-PAAs in the stock dispersion was calculated at 3.35 ± 0.18 nmol L^–1^ (mean ± standard deviation from two repeated agarose microlayer preparations).

Before titration, 300 μL of UCNP-PAA dispersion (∼7.2 mg of UCNP-PAAs) was washed with HCl 1 mmol L^–1^ protonating the surface carboxyl groups. Another addition of hydrochloric acid was then used to disperse the pellet and adjust the initial acidic pH. The nanoparticles were titrated by sodium hydroxide with phenolphthalein indicating the pink coloration of equivalence point. The consumption of NaOH for HCl titration was estimated in a blank experiment when UCNP-PAAs were not present. The estimated concentration of carboxyl groups in the UCNP-PAAs stock dispersion was 0.509 ± 0.012 mmol L^–1^ (average ± standard deviation from five repeated titrations). Finally, the number of carboxyl groups per single UCNP-PAA was estimated at 152 ± 14 thousand (mean ± standard uncertainty, Table 1). This number can be interpreted in the context of PAA structure and UCNP geometry approximated here as a 66 nm sphere. From the average molar weight of PAA (100 kg mol^–1^) and the weight of the monomer unit (72 g mol^–1^), one can estimate the number of carboxyl groups in a single PAA chain to 1388. This implies ∼ 110 PAA chains bound to a single UCNP, and 11 carboxyl groups per nm^2^ of nanoparticle surface. For a better perspective, the number of surface carboxyl groups can be related to the number of NaREF_4_ units, which compose the crystal lattice of UCNPs (RE for Y, Yb, and Tm). The number of NaREF_4_ units was ∼ 1.8 × 10^6^ per UCNP, which is one carboxyl group for each 12 NaREF_4_ units. Such calculation gives us an interesting insight into the structure of modified UCNP, which is not within the reach of, for instance, electron microscopy due to a low contrast of carbon, oxygen, and hydrogen composing the PAA chains.

### Carboxylated Polystyrene Nanoparticles

PNs are examples of intrinsically nonluminescent nanoparticles used in various applications such as biosensors^40^ or photonics,^41^ and are common model nanoparticles in ecotoxicological studies.^9^ Besides technological importance, the quantity of surface carboxyl groups is an important factor affecting their presence in the environment.^28^ The dissociation of carboxylic acid groups and subsequent introduction and increase of negative charge is associated with material properties changes such as an increase of hydrophilicity.^28^ As a result, the presence of carboxylic acid groups can affect the microplastics and nanoplastics ecological impacts, specifically dispersion, transport, and adsorption properties.^8,29,42^ This applies not only to plastics intrinsically containing these functional groups in their structures but also, for example, polyethylene, where carboxyl groups are formed at its surface by photo-oxidation weathering processes.^43^ A straightforward fluorescence labeling by protocol optimized and described previously^35^ was used to overcome the low signal-to-noise ratio impeding a direct optical microscopy counting. The PNs were swollen in the presence of THF and soaked up by hydrophobic Nile Red. Nile Red is particularly well-suited for staining polystyrene due to its strong binding, high fluorescence, poor water solubility, and negligible leakage into the aqueous phase.^8,35^ This created pink Nile-PNs with a hydrodynamic diameter of 86 nm—comparable to original nonmodified PNs with a hydrodynamic diameter of 83 nm (Figures 4a and 4b). The aqueous dispersion of Nile-PNs was stored in a refrigerator. The dispersion revealed an eye-visible reddish emission (560 nm, Figures 4c and 4d) under the excitation of a 520-nm laser beam. This fluorescence emission was not observed for the original PNs (see Figures 4c and 4d).

**Figure 4:**
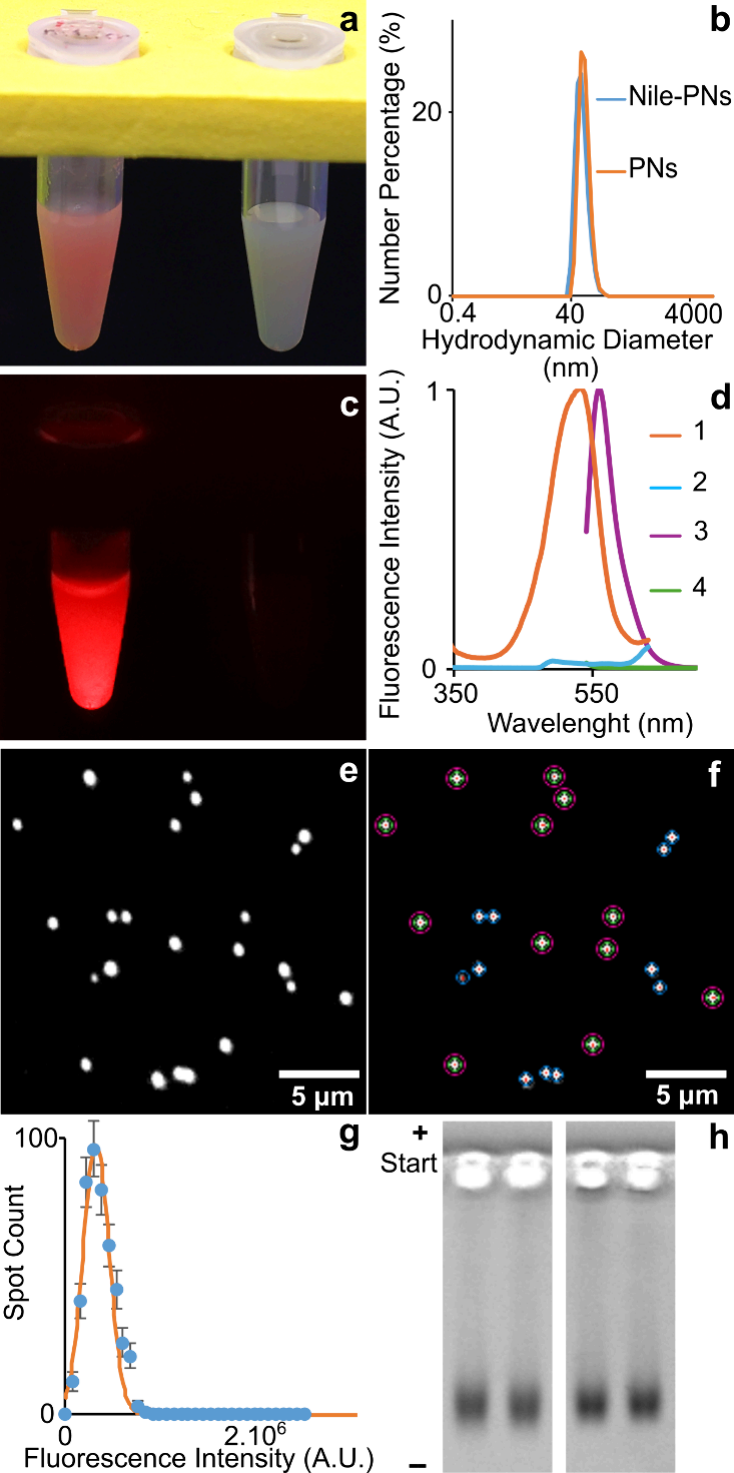
Characterization of polystyrene nanoparticles (PNs). (a) Visual appearance under white light: Nile-PNs (left) and PNs (right). (b) Size distributions of Nile-PNs (blue) and PNs (orange) measured by dynamic light scattering. (c) Fluorescence properties: Nile-PNs emit red fluorescence under 520 nm excitation, whereas PNs are nonfluorescent. (d) Spectroscopic data (nanoparticle concentration 1 nmol L^–1^): excitation spectra at 650 nm emission for Nile-PNs (spectrum 1) and PNs (spectrum 2); fluorescence spectra at 520 nm excitation for Nile-PNs (spectrum 3) and PNs (spectrum 4). (e) Fluorescence micrograph of Nile-PNs in collapsed agarose gel (520-nm excitation, 650 ± 25 nm emission, 3000 ms exposure). (f) Annotated image of panel (e): red dots indicate nanoparticle localization by U-net; green circles mark measured spot intensities; blue circles are excluded from intensity measurement; magenta circles indicate the reading of a local background. (g) Spot intensity histogram fitted with a Gaussian curve. (h) Agarose gel electrophoresis of PNs (left) and Nile-PNs (right) in 0.2% (w/w) agarose (100 V, 60 min). The gel was imaged using light transmission mode to detect light scattering by both PNs and Nile-PNs. Imaging details are provided in Note S2 and Scheme S2 in the Supporting Information).

### Analysis of Carboxylated Polystyrene Nanoparticles

To estimate the nanoparticle’s molar concentration, Nile-PNs were diluted and immobilized in the agarose gel microlayer likewise UCNP-PAAs. The microlayers were prepared in two repeats, and three micrographs were recorded for each microlayer preparation. PNs were observed as diffraction-limited spots in the fluorescence imaging mode with an emission 650 ± 25 nm band-pass filter (Figure 4e). The utilized microscope objective (magnification 100×, numerical aperture 1.30) allowed for sensitive imaging on a smaller field of view covering the area of 120 μm × 120 μm. Similarly to UCNP-PAAs analysis, the U-net localized nanoparticle spots (Figure 4f), and Python script analyzed their emission intensities (Figure 4g). A difference was in the processing of the background signal, which was a higher and quite variable compared to photon-upconversion imaging. Therefore, the background signal was measured in the local environment of each spot to correct for these irregularities. In contrast to UCNPs, the histogram of spot intensities revealed a single maximum, which was fitted well by one Gaussian curve, as shown in Figure 4g. High monodispersity of the Nile-PNs was also confirmed by gel electrophoresis (Figure 4h). Thus, the content of aggregates was considered negligible and no correction factor was used to convert between number of spots counted and the number of nanoparticles present. On average, 666 spots corresponding to discrete nanoparticles were counted per single micrograph (∼47 thousand spots per mm^2^). By considering the dilution and the thickness of the agarose microlayer before drying, the concentration of PNs in the stock dispersion was calculated to be 551 ± 19 nmol L^–1^ (mean ± standard deviation from two repeated agarose microlayer preparations).

Before titration, the PN quantity was reduced compared to UCNP-PAAs for their higher opacity. To observe the phenolphthalein color change at the equivalence point, 10 μL of PN dispersion (∼0.9 mg of PNs) was considered optimal. After washing with HCl and adjusting the initial acidic pH, the PNs were titrated with sodium hydroxide analogously to UCNP-PAAs. The estimated concentration of carboxyl groups in the PNs stock dispersion was 20.84 ± 0.65 mmol L^–1^ (average ± standard deviation from five repeated titrations). Finally, the number of carboxyl groups per single PN was estimated at 38 ± 3.6 thousand (mean ± standard uncertainty, Table 2). Similarly to UCNP-PAAs, this value can be set into the context of the geometry and the structure of nanoparticles approximated here as an 83 nm sphere. Considering the polystyrene density equal to 1 g mL^–1^ and the molar mass of the styrene unit (104 g mol^–1^), a single PN contains 1.7 × 10^6^ monomer units. This implies that there is one carboxyl group per 45 styrene units in the nanoparticle and 1.7 carboxyl groups per nm^2^ of the nanoparticle surface. As in the case of UCNPs, such calculations give us an interesting view of the PN structure.

### Limits

The quantification limits for both nanoparticle counting assay and acid–base titration should be carefully considered when setting up the analysis. For a counting assay of highly diluted samples, which may be the case of nanoplastics isolated from surface waters or other environmental samples, the concentration of nanoparticles should exceed the limit of quantification (approximately 100 fmol L^–1^) by at least an order of magnitude to enable efficient mixing with agarose. In contrast, the counting of synthesized nanoparticles is rarely limiting as stock dispersions typically contain nanoparticle concentrations in the range of 1–1000 nmol L^–1^. For the titration of carboxyl groups, experimental conditions must be meticulously optimized. Small quantities of nanoparticles yield low titration volumes, whereas excessive amounts disrupt the indicator’s color transition. The concentration of hydrochloric acid used for nanoparticle washing and titration was set to 1 mmol L^–1^. This was low enough (relative to the concentration of carboxyl groups) to enable precise titration, yet sufficiently height to ensure near-complete protonation of the PAA (p*K*_a_ ≈ 4.5).^44^ Under these conditions, approximately 3% of the carboxyl groups remained deprotonated. This led to a slight underestimation in the calculated values of carboxyl concentration. However, the resulting error was well within the measurement uncertainty and was therefore not corrected. The concentration of NaOH was chosen to be sufficiently high relative to the concentration of HCl (2.5 mmol L^–1^ used here). Increasing the amount of nanoparticles increases the number of surface carboxyl groups, which can, in principle, improve the precision of acid–base titration. However, if the nanoparticle concentration is too high, light scattering can interfere with the visual detection of the indicator color change, making it difficult to identify the titration end point. Therefore, the nanoparticle concentration was optimized to balance these effects. For UCNP-PAAs and PNs, optimal amounts of nanoparticles were 7.2 mg and 0.9 mg, respectively. Under these conditions, the limit of detection (LOD) for carboxyl groups, calculated as three standard deviations of the NaOH titration volume in blank experiments (HCl titration without nanoparticles), corresponded to 6.4 and 1.9 thousand carboxyl groups per nanoparticle for UCNP-PAAs and PNs, respectively. The limit of quantification (LOQ), defined as 10 standard deviations of the blank experiment, was determined to be 21 and 6.2 thousand carboxyl groups per nanoparticle for UCNP-PAAs and PNs, respectively. Notably, the quantities measured in this study were well above these limits (see Tables 1 and 2). The upper limit of the working range was not assessed, since samples can always be diluted to avoid exceeding the method’s capacity. The counting assay determines the concentration of nanoparticles themselves and is thus inherently independent of the specific type of surface functional group. The quantification of functional groups is performed in a separate step, allowing the approach to be adapted to a broad range of chemical functionalities. Established protocols for estimating the concentration of various functional groups are already available, as recently reviewed.^14^

## Conclusions

This study presents a robust methodology for quantifying the average number of carboxyl groups per nanoparticle by integrating acid–base titration with a single-particle counting assay in anisotropically collapsed agarose gels. The results reveal significant differences in surface functionalization between UCNP-PAAs and PNs, with UCNPs displaying a higher density of carboxyl groups per unit surface area. For UCNPs, precise quantification of surface carboxyl groups will be useful for applications such as imaging, sensing, and diagnostics, where controlled surface functionalization is essential. For PNs, this method will facilitate correlating the surface characteristics of nanoparticles with their behavior in environmental systems such as their dispersion, transport, and adsorption. Overall, this study establishes a versatile framework for analyzing nanoparticle surface chemistry, offering new opportunities for advancing nanotechnology applications and deepening our understanding of nanoparticle–environment interactions.

## Supplementary Material



## Data Availability

The data underlying this study are openly available in the ASEP data repository at 10.57680/asep.0605240.

## Notes

Image analysis; nanoparticle counting, gel electrophoresis documentation; scanner description; and gel electrophoresis of UCNP-PAAs (PDF)

## ABBREVIATIONS

*c*: molar concentration of nanoparticles
*D*: sample dilution
DMF: dimethylformamide
EDC: 1-ethyl-3-(3-(dimethylamino)­propyl)­carbodiimide
EM-CCD: electron multiplying charge-coupled device
LOD: limit of detection
LOQ: limit of quantification
MES: 2-(*n*-morpholino)­ethanesulfonic acid
*N*_A_: Avogadro’s number
NaREF_4_: sodium rare-earth fluoride
NaYF_4_/Yb^3+^/Tm^3+^: sodium yttrium fluoride doped with ytterbium and thulium ions
Nile-PNs: Nile Red-doped polystyrene nanoparticles
PAA: poly­(acrylic acid)
PNs: polystyrene nanoparticles
*R*: correction factor
Sulfo-NHS: n-hydroxysulfosuccinimide sodium salt
TEM: transmission electron microscopy
THF: tetrahydrofuran
UCNPs: photon-upconversion nanoparticles
UCNP-PAAs: photon-upconversion nanoparticles coated with poly­(acrylic acid)
U-net: convolutional neural network architecture used for image analysis
*w*: width of micrograph
