# Supplementary material for: The Application of Anisotropically Collapsing Gels, Deep Learning, and Optical Microscopy for Chemical Characterization of Nanoparticles and Nanoplastics
Source: Langmuir. 2025 May 19;41(21):13126–33. doi: 10.1021/acs.langmuir.5c00769 (PMC12139033; doi:10.1021/acs.langmuir.5c00769)
Supplement: Supplementary file 1 [file la5c00769_si_001.pdf]

# Supporting Information

## The Application of Anisotropically Collapsing Gels, Deep Learning, and Optical Microscopy for Chemical Characterization of Nanoparticles and Nanoplastics

Hana Brožková[a,b], Julie Weisová[b], Antonín Hlaváček[b]*

[a]Department of Chemistry, Faculty of Science, Masaryk University, 625 00 Brno, Czech Republic

[b]Institute of Analytical Chemistry of the Czech Academy of Sciences, Veveří 97, 602 00 Brno, Czech Republic

*Corresponding author: Antonín Hlaváček, Institute of Analytical Chemistry of the Czech Academy of Sciences, Veveří 97, 602 00 Brno, Czech Republic, E-mail: hlavacek@iach.cz

## Table of Contents

Note S1. Nanoparticle localization and counting .......... 2
Note S2. Gel Electrophoresis Scanner .......... 4
Note S3. Additional gel electrophoresis results .......... 5

# Note S1. Nanoparticle localization and counting

FIJI-ImageJ was used for visualizing and manual image evaluation. A laboratory-developed convolutional neural network was used for automatic image processing (implemented in the Python programming language using the Keras deep learning interface). The images were recorded in 16-bit pixel depth, providing intensity values from ~500 to 65535. Before neural network analysis, the images were logarithmized with a base of two and divided by 16, resulting in values from ~0.56 to 1.0. The localization of spots was performed by a convolutional neural network with a U-net architecture (Scheme S1). The U-net was selected for its already proven capability for localizing diffraction-limited spots. Following the previous reports, the model was trained on simulated data. The reasons for using simulated data are two: (A) "Ground-truth" data are not easily available for single particle localization. (B) It is possible to simulate realistic images as the physics of imaging single molecules/nanoparticles is well understood. The spots were simulated as two-dimensional Gaussian peaks with a Poisson noise. To introduce the Poisson noise, the simulated intensity was replaced with a random sample from a Poisson distribution having the mean value equivalent to that simulated intensity. The simulated peaks were superimposed on real images to introduce realistic camera noise and background. A mask indicating the positions of simulated nanoparticles was generated for each image. The size of simulated images was 128 px × 128 px. The number of 1 to 300 spots was simulated per image, and the training set contained 6400 image-mask pairs with a 20% validation split.

Once trained, the U-net processed the logarithmized and scaled images and returned maps of spot localizations, which were converted to binary masks by thresholding. The binary masks of spot localizations were used for counting nanoparticles and measuring the intensities of their luminescence by a Python script. The localization threshold was manually optimized to balance false-positive and false-negative detections in real experimental images. Due to the absence of ground-truth data, the accuracy of the U-net-based recognition could not be validated against a definitive reference. Instead, performance was assessed via expert evaluation: a human expert independently evaluated the localization results for two distinct datasets (Nile red-doped polystyrene nanoparticles; Nile-PNs, and polyacrylic acid-coated photon-upconversion nanoparticles; UCNP-PAAs) and compared them to the output of the trained U-net. For Nile-PNs, the expert identified 4001 localizations, while the U-net detected 3997, corresponding to 99.90% agreement. The false-negative rate – defined as expert-identified localizations missed by the U-net – was 0.23% (9 missed spots), and the false-positive rate – localizations identified by the U-net but not by the expert – was 0.13% (5 spurious detections). For UCNP-PAAs, the expert recorded 8079 localizations, compared to 8067 by the U-net, representing a 99.85% match. The false-negative rate was 0.22% (18 missed spots), while the false-positive rate was 0.074% (6 spurious detections). These results demonstrate high concordance between human and automated localization, supporting the reliability of the U-net model for particle recognition in the absence of ground-truth data.

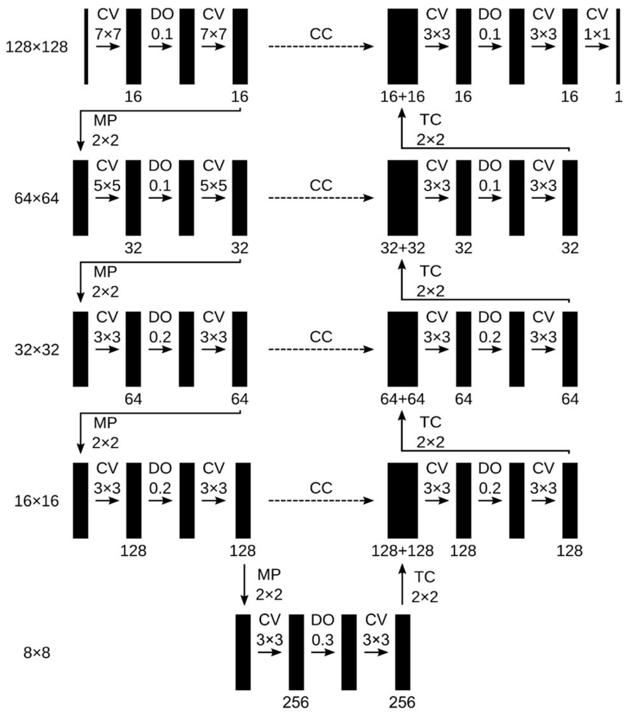


**Scheme S1**. The scheme of U-net. The U-net is composed of convolution (CV), dropout (DO), max pooling (MP), transposed convolution (TC), and concatenation (CC) layers. In the first part, the network successively reduces the size of the input image (128 px × 128 px) to a set of 256 feature maps of reduced size (8 px × 8 px). In the second part, the output image is built as a map of spot localizations (128 px × 128 px).

## Note S2. Gel Electrophoresis Scanner

To facilitate the documentation of gel electrophoresis experiments, we developed a custom-built optical scanner capable of detecting photon-upconversion emission and transmitted light. Gels were mounted on a stationary glass platform, while the optical scanning head, mounted on a motorized translation stage, traversed above the sample in three spatial axes. Optical detection was performed using a QE65 Pro CCD array spectrometer (Ocean Optics), coupled to the scanning head via a 200 µm core optical fiber (see Scheme S2 for optical setting for detecting photon-upconversion emission and transmitted light). The spectrum in the 400–850 nm range was recorded at each scanned coordinate. The scanning head was moved by stepper motors controlled by an Arduino microcontroller and a custom Java-based graphical application. This application also synchronized the spectrometer readout and head positioning. The entire apparatus was enclosed in a light-tight housing to minimize ambient light interference and maximize signal-to-noise ratio.

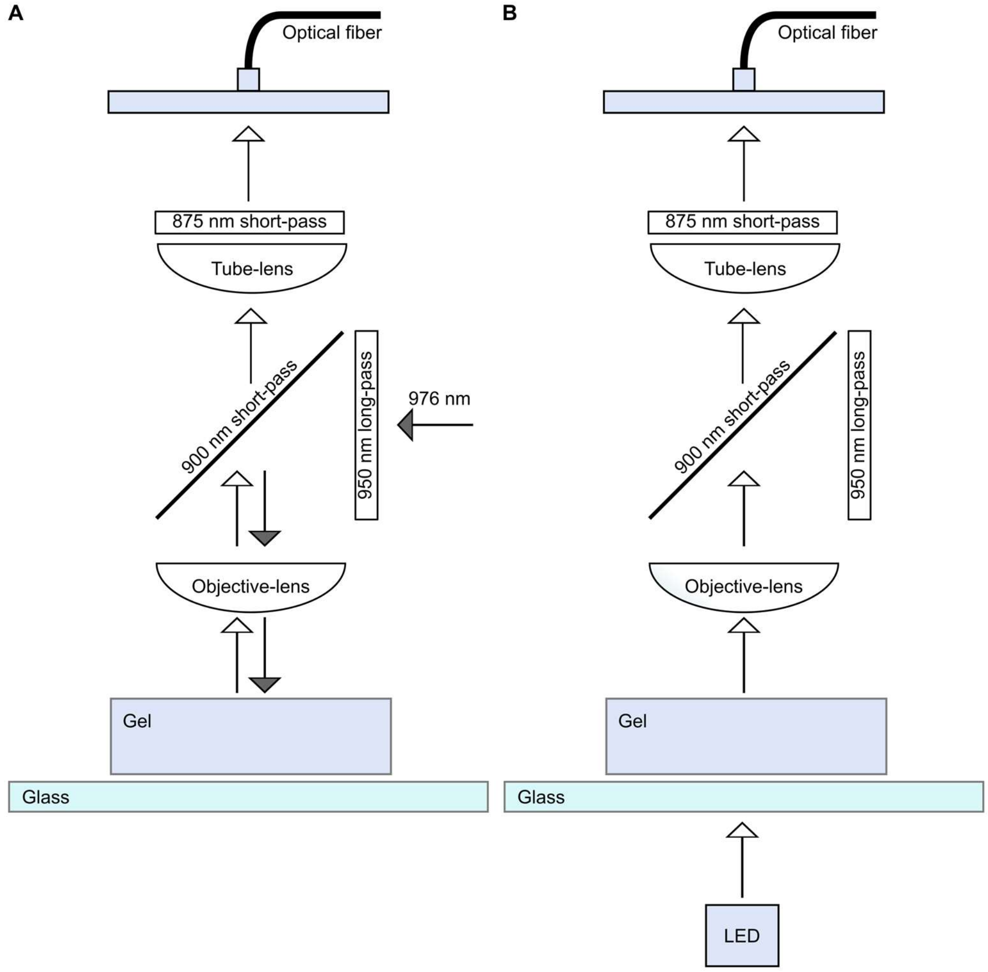


**Scheme S2.** Optical configuration of scanning heads. (A) Photon-upconversion detection. The optical scanning head employed a confocal detection geometry optimized for near-infrared excitation. A collimated 976 nm laser diode (1 W, fiber-coupled) provided the excitation light, which passed through a 950 nm long-pass filter to suppress residual shorter-wavelength components. The beam was directed toward the sample via a 900 nm short-pass dichroic mirror and focused using an achromatic doublet objective lens (focal length 30 mm, diameter 25 mm). Emission from the sample was collected by the same objective, transmitted through the dichroic mirror, and focused by a tube lens (focal length 75 mm, diameter 25 mm) onto a 200 µm core optical fiber. The fiber tip functioned as a confocal pinhole and transmitted the signal to a CCD array spectrometer. Full emission spectra (400–850 nm) were recorded at each scan point; integrated intensities within the 764–822 nm window were used as an imaging channel to construct upconversion images. (B) Transmission detection mode. For transmission imaging, the excitation laser was disconnected, and a white light-emitting diode (LED) was positioned beneath the gel. The LED moved synchronously with the scanning head, enabling uniform illumination across the sample. Light transmitted through the gel was collected using the same optical pathway as in photon-upconversion mode, including the objective lens, dichroic

mirror, short-pass filter, and tube lens. Although the dichroic mirror and the 875 nm short-pass filter remained in the optical path, they had a negligible impact on visible light transmission and were retained for operational simplicity. Full emission spectra (400–850 nm) were recorded at each scan point. The recorded spectra were integrated over two distinct wavelength ranges: 512–556 nm and 764–822 nm, here referred to as channels 1 and 2, respectively. To improve imaging quality, a ratiometric approach was applied: the intensity values in channel 1 were divided point-by-point by those in channel 2, generating channel 3. The resulting channel 3 was used as an imaging channel for gel electrophoresis documentation.

## Note S3. Additional gel electrophoresis results

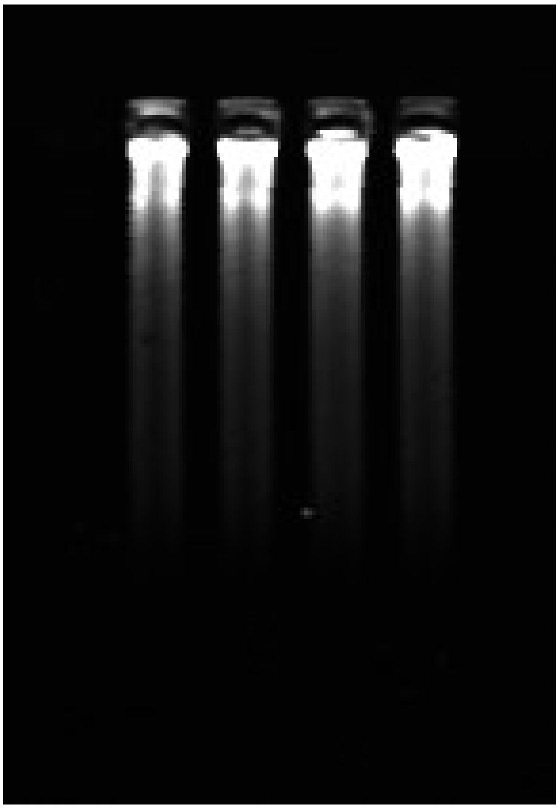

**Figure S1.** Gel electrophoresis of UCNP-PAAs. Gel electrophoresis was carried out in 0.75% (w/w) agarose gel under a voltage of 100 V for 60 min. The sample was loaded in four replicates, the concentration was ~0.1 mg $mL^{-1}$. The developed gel was scanned for a photon-upconversion with a spatial resolution of 200 μm. The excitation wavelength was 976 nm, and the detection range of emission wavelengths was 764–822 nm (see Supporting Information Note S2 for more information on scanning).
